# NMR-based metabolomic profiling reveals distinct metabolic signatures of primary hepatocytes and hepatocellular carcinoma cell lines

**DOI:** 10.1371/journal.pone.0361879

**Published:** 2026-11-12

**Authors:** Corentine Goossens, Vincent Tambay, Valérie-Ann Raymond, Marc Bilodeau

**Affiliations:** 1 CNRS-EPHE-UPVD, UAR3278, CRIOBE, Perpignan, France; 2 Laboratoire d’hépatologie cellulaire, Centre de recherche du Centre hospitalier de l’Université de Montréal (CRCHUM), Montréal, Quebec, Canada; 3 Département de médecine, Université de Montréal, Montréal, Quebec, Canada; Sun Yat-Sen University, CHINA

## Abstract

Hepatocellular carcinoma (HCC) is characterized by profound metabolic reprogramming that supports tumor initiation, growth, and metastasis. Understanding how metabolic profiles differ between normal hepatocytes and HCC cells with distinct aggressive phenotypes may help identify metabolic features associated with tumor progression. The Dt81Hepa1–6 cell line, derived from the murine Hepa1–6 hepatoma line, exhibits increased aggressiveness and provides a useful model for investigating metabolic features associated with aggressive behavior. We performed an integrative ^1^H NMR-based metabolomic analysis of intracellular extracts (endometabolome) and extracellular culture media (exometabolome) from primary hepatocytes, Hepa1–6, and Dt81Hepa1–6 cells collected at 24h and 48h. Multivariate statistical analyses, including PCA and PLS-DA, were used to characterize metabolic differences among the three cell types. Metabolic profiling revealed a clear separation between primary hepatocytes and HCC cells in both endometabolomic and exometabolomic datasets. HCC cells exhibited increased lactate and alanine levels together with decreased intracellular glucose and glutathione, consistent with altered glycolytic and redox metabolism. Time-dependent changes in glucose consumption and lactate release were prominent in extracellular media from both HCC cell lines. The aggressive Dt81Hepa1–6 line displayed a distinct metabolic phenotype characterized by increased extracellular pyroglutamate, citrate, formate and particularly BCAA-derived ketoacids, 2-oxo-isovalerate and 3-methyl-2-oxovalerate. These findings are consistent with alterations in BCAA-associated metabolism, glutamine/glutamate, and TCA-related metabolism in the more aggressive cell line. Overall, the integration of endometabolomic and exometabolomic ^1^H NMR profiling identifies metabolic features associated with the aggressive phenotype of Dt81Hepa1–6 cells. BCAA-derived ketoacids, pyroglutamate, and TCA-related metabolites emerge as candidate metabolic features that warrant validation in additional experimental and clinical HCC models.

## Introduction

Hepatocellular carcinoma (HCC) represents the most common form of primary liver cancer and remains one of the leading causes of cancer-related mortality worldwide [1,2]. Despite advances in diagnosis and treatment, the prognosis of HCC remains poor, mainly due to its high recurrence rate, metastatic potential, resistance to conventional therapies as well as the underlying liver disease that most often accompanies it [3]. A better understanding of the molecular and metabolic mechanisms underlying hepatocarcinogenesis is therefore essential to identify new biomarkers and potential therapeutic targets.

Metabolic reprogramming has emerged as a hallmark of cancer, supporting uncontrolled proliferation, survival, and invasion of malignant cells [4,5]. In liver cancer, alterations in glucose, amino acid, and lipid metabolism are particularly pronounced, reflecting both the metabolic plasticity of hepatocytes and the functional deregulation induced by oncogenic transformation [6,7]. Numerous studies have reported that hepatoma cell lines exhibit enhanced glycolytic flux (Warburg effect), altered tricarboxylic acid (TCA) cycle activity, and dependence on glutamine and branched-chain amino acid (BCAA) metabolism [8–10]. The extent to which these metabolic alterations differ between tumorigenic and non-tumorigenic cells and among HCC cell lines of varying aggressiveness remains incompletely characterized. In line with this hypothesis, there has been increasing interest in studying HCC metabolomics to better characterize its pathobiology as well as identify potential biomarkers. Recent studies have identified alterations in fatty acid metabolism as prominent features in HCC, with metabolic signatures associated with tumor progression, resistance to therapy, and the potential identification of circulating metabolic biomarkers for liquid biopsy approaches [11–14].

Nuclear magnetic resonance (NMR)-based metabolomics provides a powerful and non-destructive analytical approach for comprehensive metabolic profiling of biological systems [15,16]. Combined one- and two-dimensional NMR spectroscopy enables the identification and quantification of a broad range of metabolites within both the intracellular compartment (endometabolome) and the extracellular culture medium (exometabolome) [17]. This dual-level analysis offers a global perspective on the dynamic exchange of metabolites between cells and their environment, thereby allowing functional assessment of cellular metabolic activity under different physiological or pathological conditions [18,19].

In this context, we used an integrated ^1^H NMR-based endometabolomic and exometabolomic approach to characterize metabolic differences between murine primary hepatocytes, Hepa1–6 cells, and their more aggressive derivative Dt81Hepa1–6 [20]. Our objective was to identify discriminant metabolic features associated with tumorigenicity and increased aggressiveness within this experimental model. Multivariate statistical approaches, including Principal Component Analysis (PCA) and Partial Least Squares Discriminant Analysis (PLS-DA), were used to reveal global trends and discriminant metabolic features associated with normal, tumorigenic, and more aggressive HCC phenotypes.

## Materials and methods

### Cell lines and culture conditions

Primary hepatocytes were isolated from adult male C57BL/6 mice using the two-step collagenase perfusion method as previously described [21]. Regarding anesthesia and efforts to alleviate suffering during hepatocyte isolation, animals were kept under general anesthesia induced at 4% then kept at 2% until the end of the surgery. Animals were sacrificed by exsanguination occurring during the perfusion. The authenticated Hepa1–6 murine hepatoma cell line was obtained from the American Type Culture Collection (Manassas, VA, USA). Dt81Hepa1–6 cell line was derived from Hepa1–6 cells through *in vivo* passage in C57BL/6 mice [20]. Primary, Hepa1–6 and Dt81Hepa1–6 cells were maintained at 37°C and 5% CO_2_ and cultured in standard 25 mM glucose DMEM, supplemented with 10% Fetal Bovine Serum, 100 U/mL penicillin and 100 µg/mL streptomycin. In order to ensure 80–90% confluency after 48h for each specific cell line, 2.5 x 10^6^, 3.5 x 10^6^ and 6 x 10^6^ cells were plated in 10-cm Petri dishes for primary hepatocytes, Hepa1–6 and Dt81Hepa1–6 cells respectively.

### Experimental replication and sample collection

For each cell line, independent experimental series were initiated on separate occasions from independently thawed cryopreserved cell aliquots. Within each experimental series, cells were seeded into separate culture dishes, and each dish was processed as an individual culture sample for metabolomic analysis. For primary hepatocytes, two independent primary hepatocyte preparations were obtained from two different male mice and were cultured and analyzed separately (S1 Table).

For endometabolomic analysis, 33 primary hepatocyte extracts were analyzed, including 17 samples collected at 24 h and 16 at 48 h. These samples originated from two independent primary hepatocyte preparations. A total of 26 Hepa1–6 extracts were analyzed, including 12 samples at 24 h and 14 samples at 48 h, obtained from two independent experimental series. For Dt81Hepa1–6 cells, 27 extracts were analyzed, including 13 samples at 24 h and 14 samples at 48 h, obtained across three independent experimental series.

For exometabolomic analysis, culture medium samples were collected from the same individual culture dishes used for the corresponding endometabolomic experiments. At 24 h and 48 h, nine Dt81Hepa1–6 medium samples per time point were collected across three independent experimental series, while 12 Hepa1–6 samples per time point were collected across two independent experimental series. For primary hepatocytes, nine medium samples per time point were obtained from individual culture dishes originating from the second primary hepatocyte preparation. Additional medium samples collected at 6 h included six Dt81Hepa1–6, 12 Hepa1–6, and seven primary hepatocyte samples. A total of 22 cell-free culture medium controls were collected and analyzed in parallel with the corresponding cell culture experiments across five experimental series. The numbers of samples available for analysis varied slightly between time points because a small number of samples were lost during experimental handling and processing.

### Metabolite extraction & sample preparation

#### Growth medium.

0.3 mL of growth medium was collected at different time points (0-6-24-48 h) and mixed with 0.6 mL of cold MeOH. The samples were then centrifuged at 16 000 g at 4°C for 20 min. The protein-free supernatants were collected, dried under a gentle stream of nitrogen and stored at −80°C.

#### Cell extraction.

After 24 or 48h incubation, cell medium was removed and cells were washed with 10 mL PBS and then rapidly quenched in liquid nitrogen. The extraction of intracellular metabolites was performed on dry-ice using a dual phase CH_3_OH:H_2_O:CHCl_3_ (2:1:2) extraction procedure. Cells were scraped from the culture dishes after addition of 0.4 mL of MeOH and 0.2 mL of H_2_O and collected into a 2-mL microcentrifuge tube. Then, 0.4 mL of chloroform was added and samples were incubated at 4°C for 15 min. The solution was then centrifuged for 15 min at 16 000 g and 4°C. The supernatant containing polar metabolites was transferred to fresh tube and dried overnight under nitrogen flow. Samples were stored at −80°C until further analysis. Dried medium samples and polar extracts were dissolved in 0.6 mL of D_2_O-prepared phosphate buffer (pH 7.4) containing 0.4 mM of sodium trimethylsilyl-(2,2,3,3-d_4_)-propionate (TSP) as internal reference. Solutions were transferred to 5 mm NMR tubes for metabolite analysis.

#### NMR experiments.

NMR experiments were acquired at 298 K on a Bruker Avance II 700 MHz spectrometer equipped with a cryoprobe (5 mm CPDCH ^13^C/D-^1^H). Samples were randomized prior to NMR acquisition to minimize potential acquisition-order effects. For each sample, a one-dimensional (1D) ^1^H NMR spectrum was acquired with water signal suppression using a NOESY-presat pulse sequence, 128 transients, 65k data points, a 2.4 s acquisition time, a 4 s interpulse delay, a mixing time of 10 ms and a spectral width of 20 ppm. Shimming was optimized for each sample using the TSP resonance, with a maximum acceptable full width at half maximum (FWHM) of 1 Hz. Spectral quality and consistency were additionally monitored using the TSP reference signal, with the coefficient of variation (CV) of its signal-to-noise ratio across samples maintained below 10%. Free Induction Decays (FID) were multiplied by an exponential function equivalent to a 0.3 Hz line-broadening factor before Fourier transformation. Resulting 1D spectra were phased, baseline corrected and referenced to the TSP signal at 0 *ppm* using TopSpin 4.0.5 (Bruker).

#### Data analysis.

In order to simplify data analysis, 1D spectra were binned in the range between 0.5 and 9.5 *ppm* by intelligent bucketing, and the corresponding spectral areas were integrated using NMRProcFlow open-source software. The region between 4.5–5 *ppm,* corresponding to residual solvent, was removed. The total spectra areas were calculated from the remaining buckets and used for normalization. Metabolite profiles were analyzed using a combination of univariate and multivariate statistical methods with BioStatFlow v.2.9.2., MetaboAnalyst 5.0 and Prism (GraphPad Software, La Jolla, CA). Variables were mean-centered and divided by the standard deviation of each variable prior to statistical analysis. Principal component analysis (PCA) was first performed to enable global visualization of natural clusters and to detect potential outliers. Partial least squares-discriminant analysis (PLS-DA) was used to assess differences in metabolic phenotypes among cell types. PLS-DA analyses were restricted to the 24 h and 48 h time points, for which both endometabolomic and exometabolomic data were available, allowing consistent comparison of intracellular and extracellular metabolic profiles. The samples collected at 6 h of cell culture, available only for the exometabolomic dataset, were retained for the extracellular time-course analysis but were not included in the supervised multivariate analyses. PLS-DA model performance was assessed using Leave-One-Out Cross-Validation (LOOCV), with goodness of fit (R^2^) and predictive ability (Q^2^) as performance metrics. Permutation testing (1000 permutations of class labels) was used to assess whether PLS-DA model performance was significantly better than expected by chance, with empirical p-values calculated from the proportion of permuted models achieving performance equal to or greater than that of the original model. Individual metabolite differences among cell types were assessed using one-way analysis of variance (ANOVA), followed by Fisher’s least significant difference (LSD) post-hoc test for pairwise comparisons. For the time-course analysis of extracellular metabolites, the effects of cell type, time, and their interaction were assessed using two-way ANOVA. P-values were adjusted for multiple testing using Benjamini-Hochberg false discovery rate (FDR) and FDR < 0.05 was considered statistically significant. In addition to statistical significance, effect sizes were quantified using η^2^ for one-way ANOVA and ηp^2^ for the main effects and interaction in two-way ANOVA. Two-sided 95% confidence intervals (CI) for η^2^ and ηp^2^ were estimated using the noncentral F-distribution method.

#### Metabolite identification.

Metabolite assignment was based on ^1^H chemical shifts, signal multiplicities and spin-coupling patterns, supported, where applicable, by 2D ^1^H-^1^H TOCSY experiments, using Chenomx NMR suite software (Chenomx, Inc., Edmonton, AB, Canada), and in comparison with established NMR databases, including the Human Metabolome Database (HMDB) and Biological Magnetic Resonance Data Bank (BMRB). As no authentic reference standards were analyzed under identical experimental conditions, all reported metabolites were conservatively classified as MSI Level 2 (putatively annotated compounds) according to the Metabolomics Standards Initiative criteria.

## Results

### ^1^H NMR analysis of cell extracts and growth media

Cell extracts and growth media were analyzed after 24 h and 48 h of incubation to assess the potential of metabolic profiling for comparing and characterizing the metabolic activity of HCC cell lines and primary hepatocytes. Fig 1 presents the 700 MHz ^1^H NMR spectra of cell extracts and culture media from primary hepatocytes (PH), Hepa1–6 (H1-6), and Dt81Hepa1–6 (DT) cell lines. Table 1 summarizes the metabolites detected in both intracellular (E, extracts) and extracellular (M, medium) compartments.

**Table 1:** Metabolites detected by ^1^H NMR in culture media and polar cell extracts. Chemical shifts and signal multiplicities used for metabolite annotation are reported together with their detection in culture medium (M) and/or cellular extracts (E). All metabolite assignments were classified as MSI Level 2 (putatively annotated compounds).

Metabolite	^1^H NMR shifts (ppm)	Sample
**α-ketoacids and derivatives**		
2-oxoisocaproate	0.94 (d), 1.62 (m), 2.22 (m), 2.66 (m)	M
2-oxoisovalerate	0.94 (d), 1.01 (d), 2.22–2.30 (m), 2.60–2.66 (m)	M
3-hydroxybutyrate	1.20 (d), 2.31 (dd), 4.15 (m)	M
3-hydroxyisobutyrate	1.17 (s), 2.47 (d), 3.61 (m)	M
3-methyl-2-oxovalerate	0.94 (d), 1.13 (d), 2.17–2.35 (m)	M
4-hydroxybutyrate	2.01 (m), 2.37 (t), 3.68 (t)	M
**Nucleotides and cofactors**		
AMP	6.15 (d), 8.26 (s), 8.57 (s)	E
ATP	6.15 (d), 8.25–8.60 (m)	E
Creatine/PCr	3.04 (s), 3.92 (s)	E
NAD^+^	6.02 (d), 8.18 (d), 8.94 (s), 9.33 (s)	E
UDP-species	4.00–4.35 (m), 5.97 (d), 7.90 (s), 8.11 (s)	E
**Organic acids**		
Acetate	1.92 (s)	M,E
Acetone	2.23 (s)	M
Citrate	2.53 (d), 2.69 (d)	M,E
Formate	8.46 (s)	M
Malate	2.34 (dd), 2.66 (dd), 4.29 (m)	E
Pyruvate	2.37 (s)	M
Succinate	2.41 (s)	M,E
Lactate	1.32 (d), 4.11 (q)	M,E
**Amino acids**		
Alanine	1.48 (d), 3.78 (q)	M,E
Arginine	1.68 (m), 1.92 (m), 3.23 (m)	M,E
Aspartate	2.68 (dd), 2.80 (dd), 3.89 (m)	E
ß-Alanine	2.57 (t), 3.17 (t)	E
Glutamate	2.07 (m), 2.35 (m), 3.75 (dd)	E
Glutamine	2.13 (m), 2.45 (m), 3.77 (m)	M,E
Glycine	3.55 (s)	M,E
Histidine	7.05 (s), 7.92 (s)	M,E
Isoleucine	0.93 (t), 1.01 (d), 1.27 (m), 3.67 (d)	M,E
Leucine	0.96 (d), 0.97 (d), 1.72 (m)	M,E
Lysine	1.47 (m), 1.75 (m), 3.01 (t)	M,E
Methionine	2.13 (s), 2.63 (m), 3.82 (t)	M,E
Phenylalanine	7.32–7.43 (m), 3.14 (dd), 3.29 (dd)	M,E
Proline	1.98 (m), 2.36 (m), 3.34 (m), 4.12 (m)	M,E
Serine	3.83 (dd), 3.98 (dd)	M,E
Threonine	1.33 (d), 3.58 (m), 4.23 (m)	M,E
Tryptophan	7.20–7.75 (m), 3.29 (m), 4.05 (m)	M
Tyrosine	6.88 (d), 7.18 (d), 3.05 (dd)	M,E
Valine	0.98 (d), 1.03 (d), 2.27 (m)	M,E
**Nitrogenous compounds, osmolytes, derivatives**		
Betaine	3.26 (s)	M,E
Choline	3.20 (s), 3.51 (dd), 4.05 (m)	M
Glycerophosphocholine (GPC)	3.22 (s), 3.62 (m), 4.30 (m)	E
O-Phosphocholine (O-PC)	3.23 (s), 4.15 (m)	E
Glutathione (GSH)	2.15 (m), 2.55 (m), 2.95 (dd), 3.77 (m)	E
Hypotaurine	2.66 (t), 3.36 (t)	E
Taurine	3.27 (t), 3.43 (t)	E
Myo-Inositol	3.28–3.63 (m)	E
Ornithine	1.70 (m), 1.90 (m), 3.03 (t)	E
**Sugars and intermediates**		
Glucose	3.23–3.90 (m), 5.23 (d)	M,E
Glucose-6-phosphate	3.45–4.05 (m), 5.25 (d)	E
**Others**		
Ethanol	1.18 (t), 3.65 (q)	M
Niacinamide	7.54 (d), 8.70 (d), 9.14 (s)	M
Propionate	1.05 (t), 2.18 (q)	M
Pyridoxine	2.64 (s), 2.81 (s), 4.82 (s), 7.75 (s)	M
Pyroglutamate	2.15 (m), 2.40 (m), 4.30 (dd)	M,E

**Fig 1:**
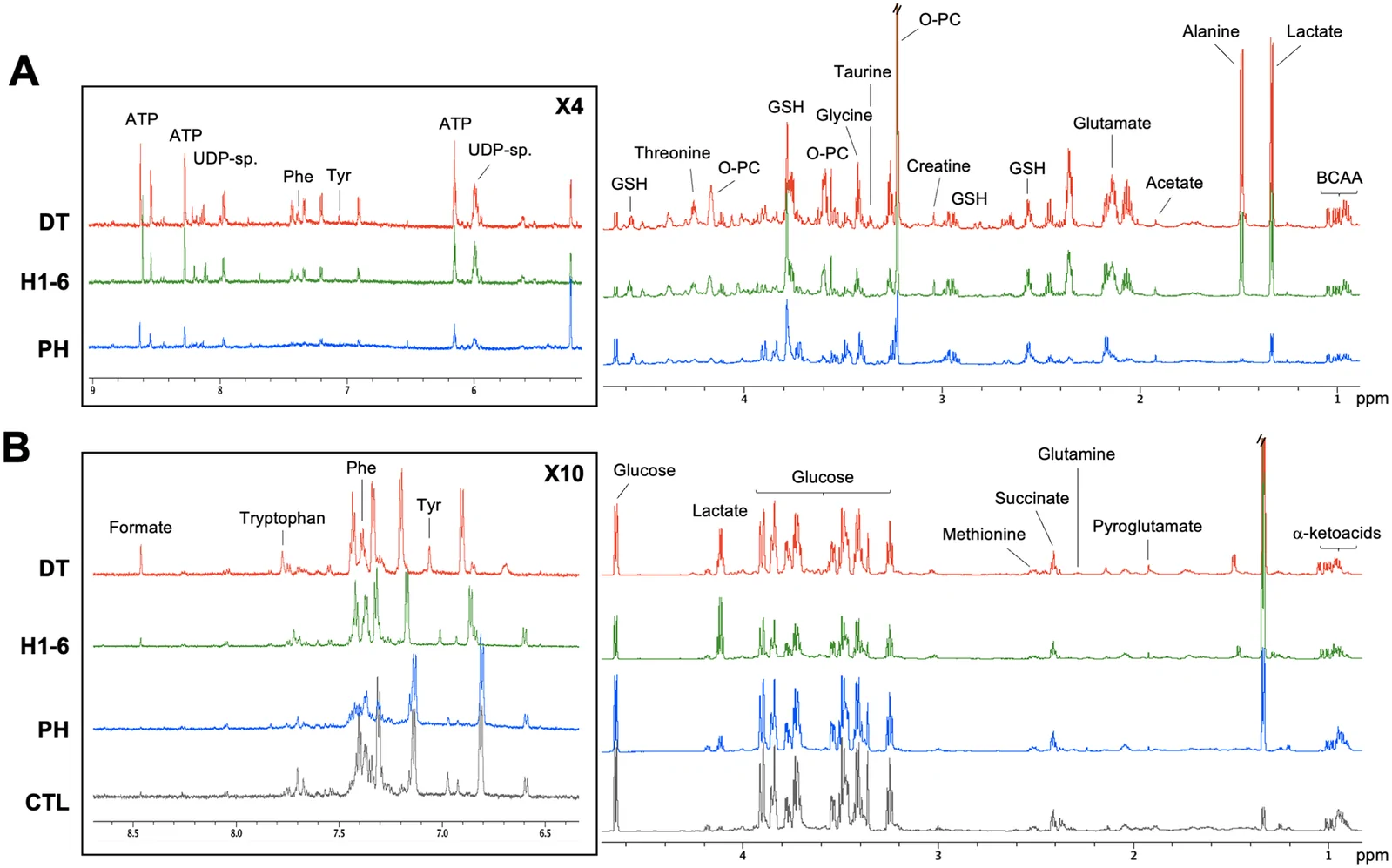
Representative 700 MHz ^1^H NMR spectra (δ1-δ4.5 ppm and expanded δ6-δ9 ppm regions) of (A) polar cell extracts and (B) culture media collected after 48 h from each cell type. PH, primary hepatocytes; H1-6, Hepa1-6; DT, Dt81Hepa1-6; Ctl, cell-free DMEM supplemented with 10% FBS. BCAA, branched-chain amino acids; GSH, glutathione; O-PC, O-phosphocholine; UDP-sp., UDP-species.

### Exploratory data analysis

Unsupervised Principal Component Analysis (PCA) was first applied to the NMR datasets to identify major sources of variance and detect potential outliers. The PCA score plots of polar metabolites from cell extracts (endometabolome) and culture media (exometabolome) are shown in Figs 2A and 2B, respectively, for primary hepatocytes and the two HCC cell lines, Dt81Hepa1–6 and Hepa1–6.

**Fig 2:**
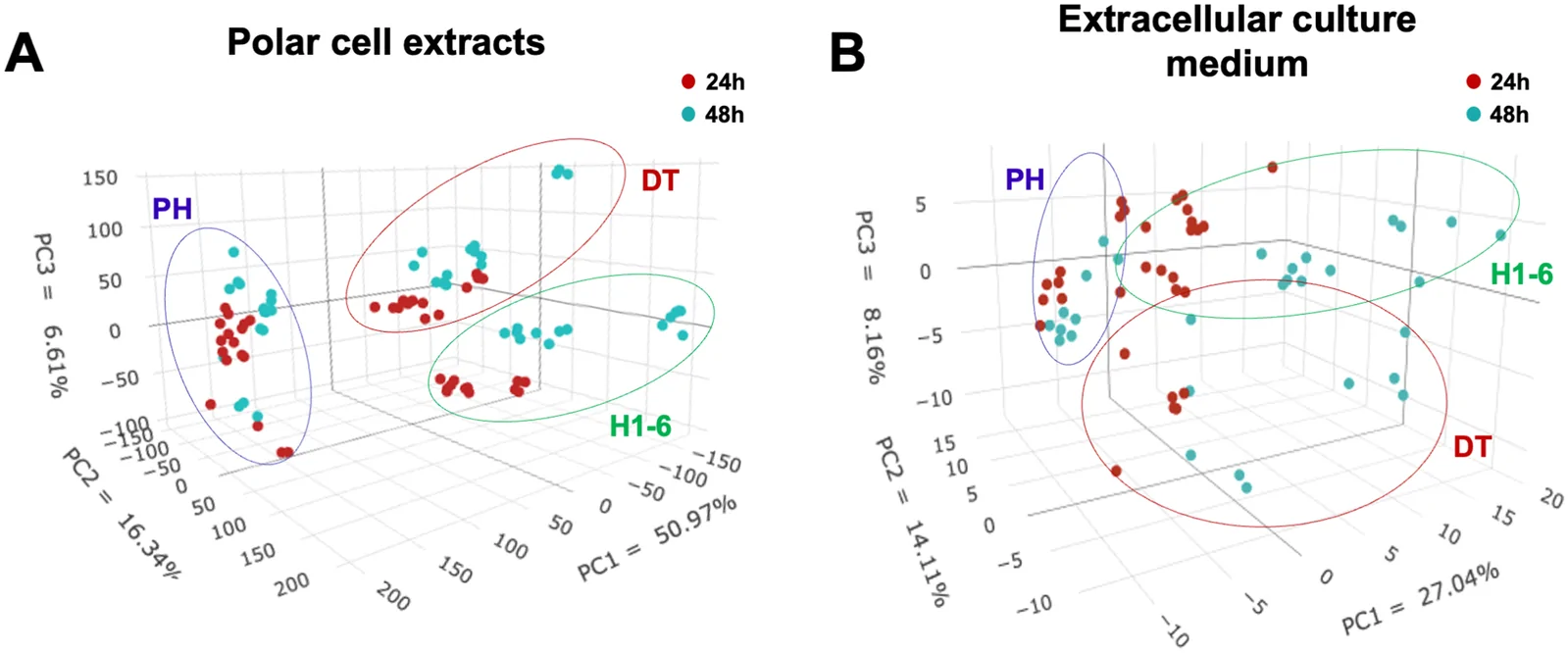
Scores plots obtained from unsupervised principal component analysis (PCA) of ^1^H NMR data from (A) polar cell extracts and (B) culture media. Samples are grouped according to cell type (PH, primary hepatocytes; H1-6, Hepa1-6; DT, Dt81Hepa1-6) and collection time (24 h and 48 h).

For cell extract data, PC1 and PC2 accounted for 51% and 16% of the total variance, respectively, whereas for culture media they accounted for 27% and 14%, respectively (Fig 2A,B). A clear separation between tumorigenic and non-tumorigenic profiles was observed along PC1 for both endometabolomic and exometabolomic datasets. Within each cellular group, further sub-clustering along PC1 corresponded to the two incubation times (24 h and 48 h). Additionally, separation between Dt81Hepa1–6 and Hepa1–6 samples was evident along PC2 and PC3. The corresponding biplots indicating the metabolites contributing to these separations are shown in S1 Fig.

To further characterize metabolic differences among the three cell types, supervised PLS-DA models were performed on the endometabolome and exometabolome datasets. Importantly, the separation observed in the supervised PLS-DA models was consistent with the group structure already apparent in the corresponding unsupervised PCA analyses.

### Intracellular metabolites differentiating HCC and primary cells

To characterize the intracellular metabolic profiles of HCC cells and primary hepatocytes, a PLS-DA model was constructed (Fig 3A). The two-component model yielded R^2^ = 0.97 and Q^2^ = 0.97 and was evaluated using leave-one-out cross-validation (LOOCV) and a 1000-fold permutation test (p < 0.001; S2B Fig).

**Fig 3:**
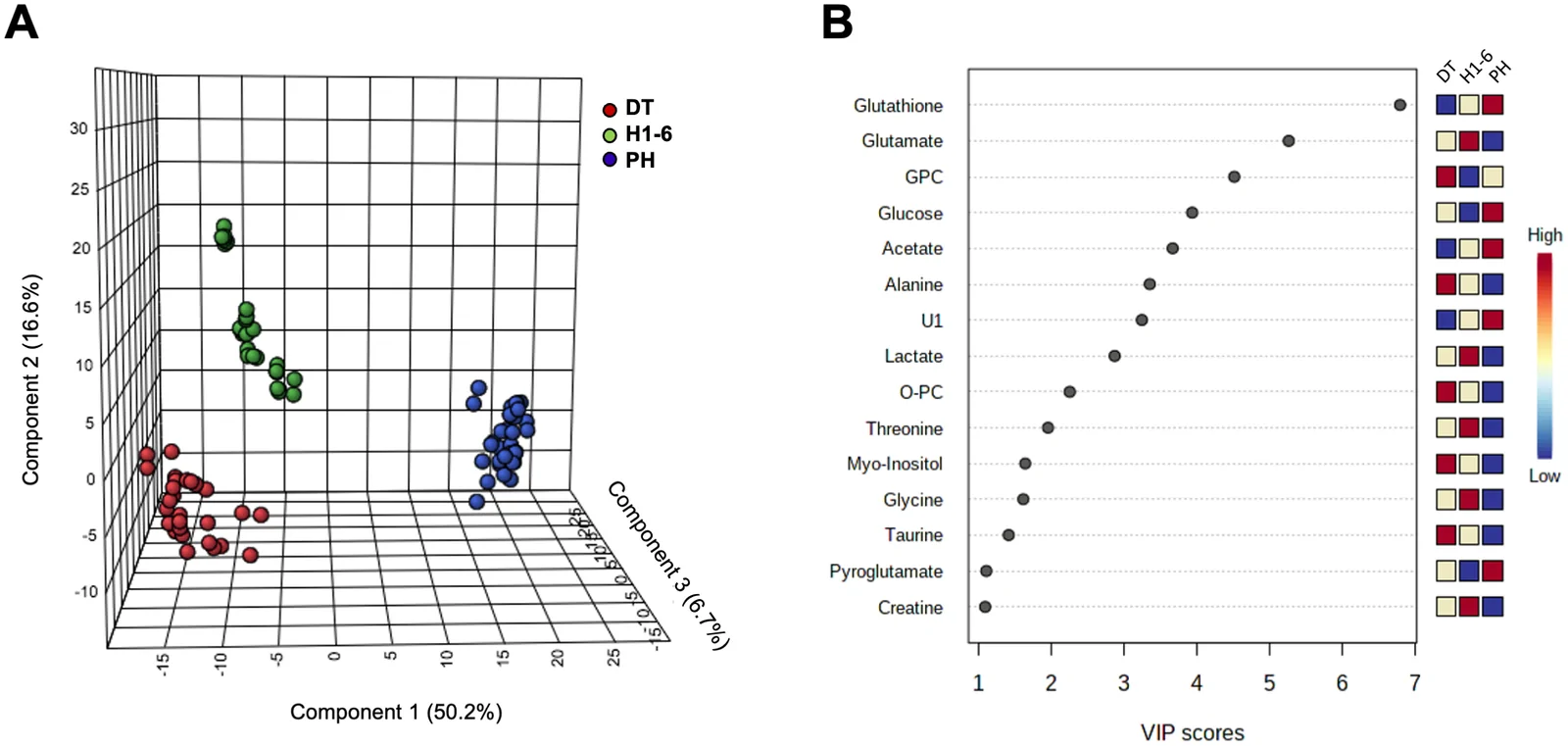
PLS-DA of the endometabolic profiles of primary hepatocytes, Hepa1-6 and Dt81Hepa1-6 cells. PLS-DA was performed on ^1^H NMR data from polar cell extracts collected at 24 h and 48 h. (A) PLS-DA score plot showing the distribution of Dt81Hepa1-6 (DT), Hepa1-6 (H1-6), and primary hepatocyte (PH) samples. (B) Variable Importance in Projection (VIP) score plot showing the metabolites contributing most strongly to group discrimination. Colored boxes indicate the relative abundance of each metabolite across the three cell types.

Variable Importance in Projection (VIP) scores identified the variables contributing most strongly to the discrimination between cell types (Fig 3B). The 15 metabolites with the highest VIP scores were further examined by univariate ANOVA (Fig 4). Cell-type effects were significant for all 15 metabolites after FDR correction (S2 Table), with η^2^ values ranging from 0.347 to 0.938. Glutamate, glutathione, glucose and Unknown1 showed particularly large cell-type effects (η^2^ = 0.938, 0.889, 0.884 and 0.881, respectively). Overall, glutamate, alanine, lactate, threonine, myo-inositol, glycine, taurine, and creatine levels were higher in HCC cells than in primary hepatocytes, whereas glucose, glutathione, acetate, and pyroglutamate were lower. Choline compounds also differed among cell types, with GPC and O-PC particularly elevated in Dt81Hepa1–6 cells.

**Fig 4:**
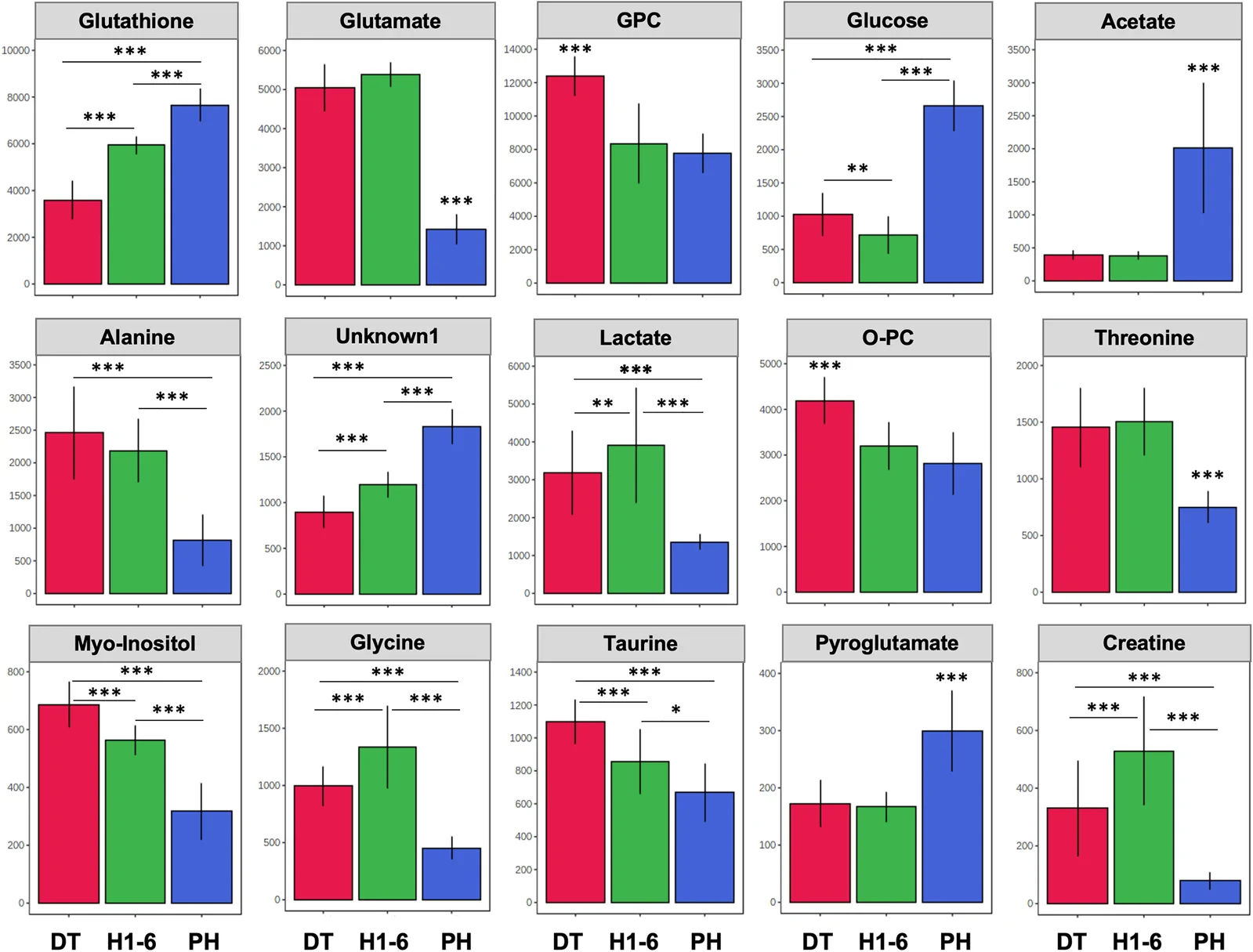
Relative levels of the 15 intracellular metabolites with the highest VIP scores. Metabolite levels are shown for Dt81Hepa1-6 (DT), Hepa1-6 (H1-6), and primary hepatocyte (PH) cell extracts. Data are presented as mean ± SD. Differences among cell types were assessed by one-way ANOVA followed by Fisher’s LSD post-hoc test. Detailed ANOVA statistics, FDR-adjusted p-values, effect sizes (η^2^), 95% confidence intervals and post-hoc comparisons are provided in S2 Table. * p < 0.05; ** p < 0.01; *** p < 0.001.

### Extracellular metabolites differentiating HCC and primary cells

To obtain an overview of extracellular metabolic over time, a PCA model was generated using culture medium samples collected at 0, 6, 24, and 48 hours from cell-free control medium (Ctl; DMEM supplemented with 10% FBS), primary hepatocytes, Hepa1–6, and Dt81Hepa1–6 cells (Fig 5A). PC1 revealed a clear temporal change in HCC media, indicating that incubation time was the primary source of variance.

**Fig 5:**
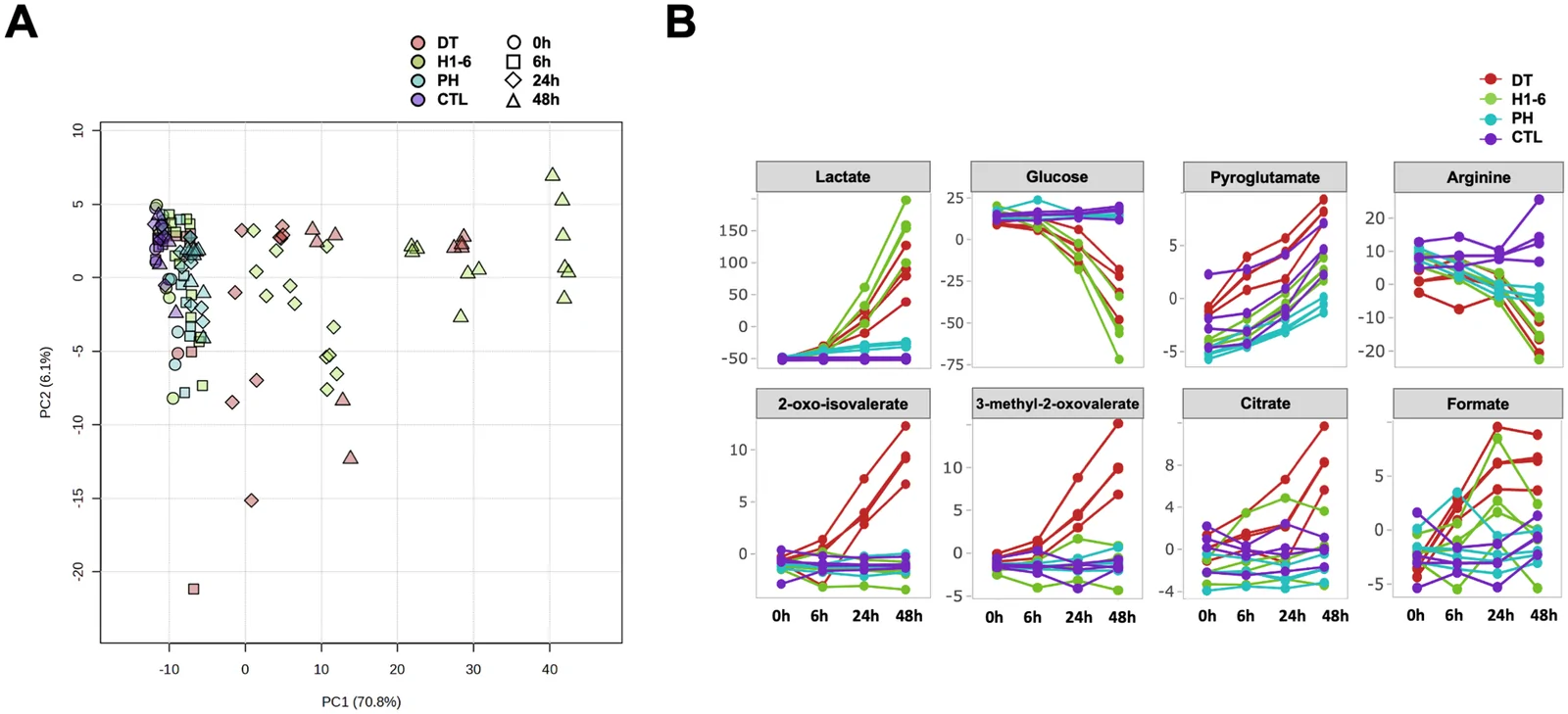
Time-dependent changes in extracellular metabolic profiles. (A) PCA scores plot of culture media collected at 0, 6, 24, and 48 h from cell-free control medium (Ctl; DMEM supplemented with 10% FBS), primary hepatocytes (PH), Hepa1-6 (H1-6) and Dt81Hepa1-6 (DT) cultures. Symbols indicate sampling time (circle, 0 h; square, 6 h; diamond, 24 h; triangle, 48 h), and colors indicate experimental groups. (B) Time-course profiles of selected extracellular metabolites. Effects of cell type, time, and their interaction were assessed by two-way ANOVA with Benjamini-Hochberg FDR correction. Detailed FDR-adjusted p-values, effect sizes (ηp^2^), and 95% confidence intervals are provided in S3 Table. Statistical significance was defined as FDR < 0.05.

Two-way ANOVA revealed significant effects of cell type and time on the extracellular levels of the metabolites displayed in Fig 5B, with substantial effect sizes for several metabolites. Significant TYPE x TIME interactions were also observed for most metabolites, indicating cell-type-dependent temporal metabolic responses. In particular, HCC cells showed higher consumption of glucose and arginine and increased release of lactate and pyroglutamate compared with control and primary hepatocyte media. Interestingly, 2-oxo-isovalerate and 3-methyl-2-oxovalerate showed a marked and time-dependent accumulation in Dt81Hepa1–6 media, whereas their levels remained low in the other groups. Detailed FDR-adjusted p-values, ηp^2^ effect sizes, and 95% confidence intervals are provided in S3 Table.

To further examine the exometabolome profiles, supervised PLS-DA models were computed for the 24 h and 48 h culture media (S2 Fig). The model exhibited strong predictive ability for discriminating between cell types (R^2^ = 0.90, Q^2^ = 0.83, 2 components) and was validated by LOOCV and a 1000-fold permutation test (S4 Fig). VIP analysis identified the 15 variables contributing most strongly to the discrimination between cell types (Fig 6A).

**Fig 6:**
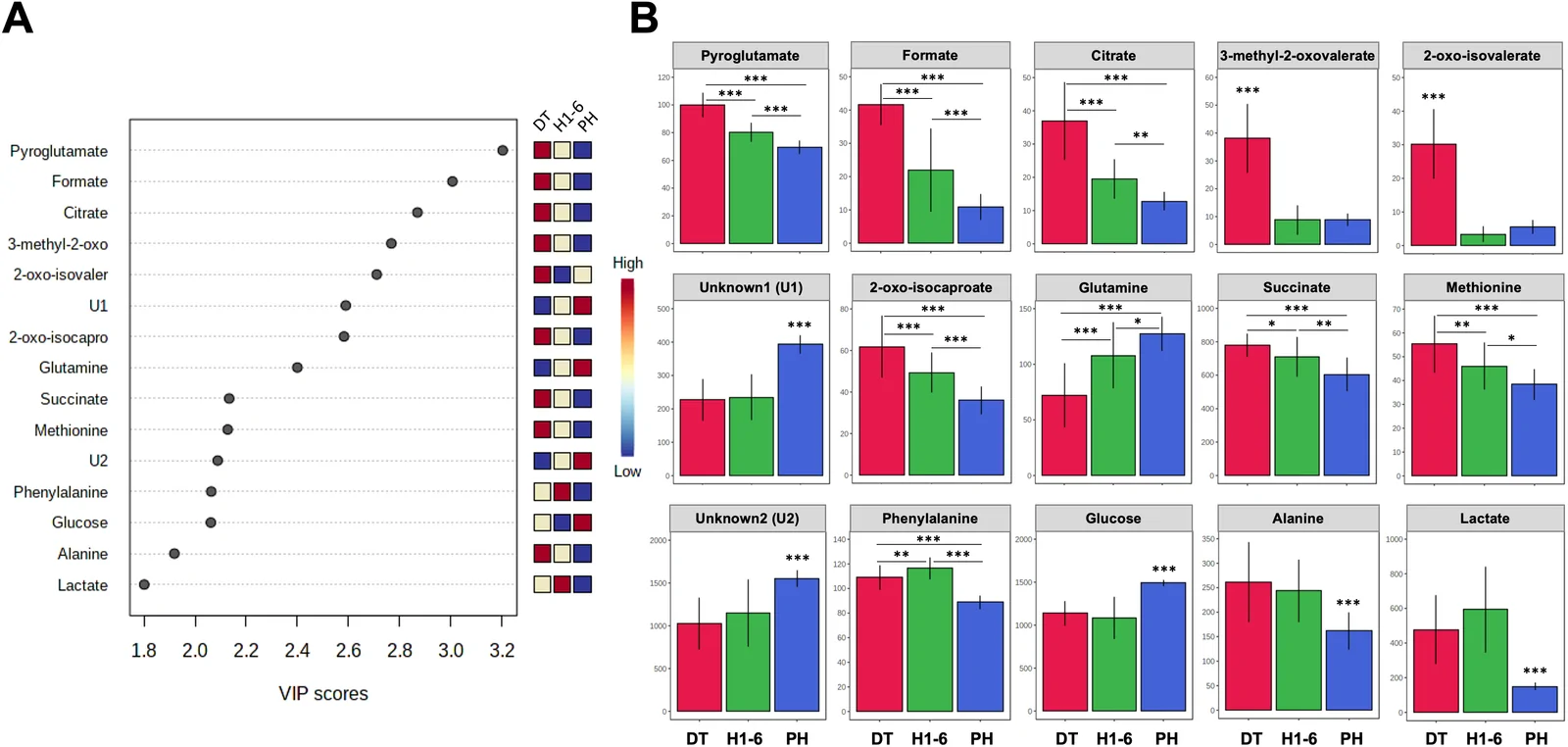
Extracellular metabolites contributing most strongly to discrimination among cell types. (A) Variable Importance in Projection (VIP) score plot derived from the PLS-DA of culture media from Dt81Hepa1-6 (DT), Hepa1-6 (H1-6), and primary hepatocyte (PH) cultures collected at 24 h and 48 h. Colored boxes indicate the relative abundance of each metabolite across the three groups. (B) Relative levels of the 15 extracellular metabolites with the highest VIP scores. Data are presented as mean ± SD. Differences among groups were assessed by one-way ANOVA followed by Fisher’s LSD post-hoc test. Detailed ANOVA statistics, FDR-adjusted p-values, effect sizes (η^2^), 95% confidence intervals, and post-hoc comparisons are provided in S4 Table. * p < 0.05; ** p < 0.01; *** p < 0.001.

Univariate ANOVA of these 15 VIP metabolites revealed significant differences among cell types after FDR correction (Fig 6B and S4 Table), with effect sizes ranging from η^2^ = 0.308 to 0.809. Pyroglutamate, formate, citrate, 3-methyl-2-oxovalerate, and 2-oxo-isovalerate were significantly higher in Dt81Hepa1–6 media than in Hepa1–6 and primary hepatocyte media and showed particularly large cell-type effects (η^2^ = 0.756, 0.663, 0.634, 0.764, and 0.809, respectively). In addition, 2-oxo-isocaproate, succinate, methionine, phenylalanine, alanine, and lactate were higher in HCC media, whereas glutamine and glucose were lower compared with primary hepatocyte media. Detailed ANOVA statistics, FDR-adjusted p-values, η^2^ effect sizes with 95% confidence intervals, and Fisher’s LSD post-hoc comparisons are provided in S4 Table.

## Discussion

The present study provides an in-depth comparison of the metabolic profiles of primary hepatocytes and two HCC cell lines, Hepa1–6 and its more aggressive derivative Dt81Hepa1–6, using an NMR-based metabolomic approach. By integrating intracellular and extracellular metabolite data, we identified specific metabolic signatures associated with tumorigenicity and aggressiveness in liver cancer cells.

### Metabolic reprogramming distinguishes HCC cells from primary hepatocytes

The clear segregation of tumorigenic and non-tumorigenic cells in PCA and PLS-DA models highlights marked differences in their metabolic profiles. Consistent with the “Warburg effect,” HCC cells exhibited enhanced glycolytic activity, reflected by elevated intracellular and extracellular lactate levels and a concomitant reduction in glucose concentrations in both the endometabolome and exometabolome [4,5]. In parallel, increased alanine levels are consistent with altered handling of glycolysis-derived pyruvate through transamination reactions catalyzed by alanine aminotransferase. This association between glycolysis and amino acid metabolism may provide an alternative route for pyruvate utilization while contributing to cellular carbon and nitrogen balance in proliferating cancer cells. Elevated choline compounds further point to increased membrane lipid turnover, another hallmark of rapidly dividing cells [6,19,22].

A significant decrease in glutathione and acetate levels in tumor cells compared with primary hepatocytes is consistent with alterations in redox homeostasis and central carbon metabolism [23]. In contrast, higher levels of taurine, creatine, and myo-inositol may reflect alterations in osmotic regulation and cellular energy homeostasis associated with the transformed phenotype [10,24].

### Distinct metabolic traits of the aggressive Dt81Hepa1–6 line

Beyond the shared cancerous phenotype, Dt81Hepa1–6 displayed a distinctive metabolic fingerprint compared to its parental Hepa1–6 line, consistent with its higher aggressiveness. The selective accumulation of 2-oxo-isovalerate and 3-methyl-2-oxovalerate in Dt81Hepa1–6 culture medium indicates altered BCAA-associated metabolism in this aggressive cell line. Although extracellular accumulation of these BCAA-derived ketoacids does not by itself demonstrate increased intracellular BCAA catabolic flux, it may reflect changes in BCAA transamination, downstream ketoacid oxidation, mitochondrial metabolism, or metabolite transport and export. Given the established roles of BCAA metabolism in carbon and nitrogen homeostasis, such alterations may contribute to metabolic flexibility in highly proliferative cells [9,25]. Direct isotope-tracing and flux analyses will nevertheless be required to determine whether increased BCAA catabolism contributes functionally to the aggressive phenotype observed in Dt81Hepa1–6 cells.

The higher release of pyroglutamate, formate, and citrate observed in Dt81Hepa1–6 media is consistent with alterations in glutamine/glutamate and TCA-associated metabolism [10,23,26]. Notably, pyroglutamate (5-oxoproline) was selectively increased in the extracellular medium of Dt81Hepa1–6 cells. Pyroglutamate is an endogenous metabolite linked to glutamate metabolism and the γ-glutamyl cycle involved in glutathione turnover and redox homeostasis. Its increased extracellular abundance, together with the lower intracellular glutathione levels observed in tumor cells, may therefore be consistent with altered glutamate/glutathione metabolism and redox homeostasis [27,28]. With regards to glutamine metabolism, we have previously studied the reprogramming of glutaminase in HCC, in which we showed increased glutamine utilization as a hallmark of HCC tumors compared to cirrhotic and normal liver [29]. The finding of decreased extracellular glutamine over the course of cell culture reported within this study provides further evidence for the hypothesis of glutamine addiction in HCC, in which HCC cells are avid consumers of exogenous glutamine.

Furthermore, in line with our identification of metabolic signatures associated with HCC cell aggressiveness, previous studies have also reported associations between specific metabolic alterations and aggressive HCC phenotypes. Notably, two recent studies identified lipid-related metabolites, including free fatty acids, as predictors of HCC aggressiveness and progression in patient samples [30,31]. Although focused on lipidomics, these findings, taken with ours, underscore that metabolomic signatures differ according to HCC phenotype and may indeed have prognostic value.

### Endo- and exometabolome integration as a complementary approach

Better understanding the tumor microenvironment in HCC and other cancers has gained interest in recent years. As metabolites represent key intermediates linking cancer cells with their extracellular environment, we simultaneously analyzed intracellular and extracellular metabolomes to investigate how HCC cells modify their extracellular environment in parallel with intracellular metabolic alterations. The parallel changes observed in glucose and lactate across the two compartments support the hypothesis of dynamic metabolite flux between cells and their environment. Moreover, exometabolomic analysis revealed pronounced time-dependent changes, particularly in HCC cells, emphasizing the importance of culture duration when assessing extracellular metabolic phenotypes [15,16,18]. Interestingly, a recent study demonstrated that HCC cells exhibit increased vulnerability to glycolysis inhibition under hypoxia, highlighting that specific microenvironmental conditions can shape cancer cell metabolic reprogramming and influence the uptake and release of nutrients and metabolic byproducts [32]. The characterization of the exometabolome is particularly relevant in the context of liquid biopsy, where the identification of circulating biomarkers, including metabolites, represents an ideal means for improving the detection and characterization of HCC. In fact, recent work has highlighted the potential for circulating plasma metabolites in prediction of HCC diagnosis and progression [33,34].

### Biological and translational implications

Overall, Dt81Hepa1–6 cells displayed a more pronounced metabolic divergence from primary hepatocytes than the parental Hepa1–6 line, identifying a set of metabolic features associated with the aggressive phenotype within this experimental model. In particular, BCAA-derived ketoacids, pyroglutamate, and citrate represent candidate discriminant metabolites that warrant further investigation in independent HCC models of varying aggressiveness, particularly through isotope-tracing of metabolites *in vitro* and *in vivo*. The findings presented herein are consistent with previous findings from our group, which reported significantly distinct metabolic profiles between HCC tissues and adjacent normal liver in patient samples, supporting the relevance of our cellular model [35]. Although direct comparison between the current findings and our previous study is limited, given the extent of differences in methodological design of both studies, we note that increased glutamate was observed in human HCC tissues as well as HCC cell endometabolomes. Notably, decreased intracellular glutathione was consistently observed across the two experimental settings. Glutathione also represented an important discriminating feature in our previous analysis and was among the metabolites with high VIP scores in the present study [35]. These concordant alterations suggest that metabolic pathways, including redox metabolism, identified through *in vitro* analyses have translational relevance. However, the integration of endometabolomic and exometabolomic profiles provides a complementary framework for investigating metabolic alterations and metabolite dynamics between cancer cells and their extracellular environment, which may contribute to tumor progression in other experimental cancer models [36–38].

### Study limitations and future perspectives

Several limitations should be considered when interpreting the present findings. First, ^1^H NMR-profiling provides information on relative metabolite abundance but does not directly measure metabolic flux. Accordingly, the observed changes in BCAA-derived ketoacids, glutamine/glutamate-related metabolites, and TCA-associated metabolites should be interpreted as metabolic signatures rather than direct evidence of altered pathway flux. As discussed above, isotope-tracing and functional studies will be required to establish the underlying mechanisms and their contribution to the aggressive phenotype of Dt81Hepa1–6 cells, in both the current *in vitro* model and its corresponding *in vivo* model.

Second, the present study was conducted using a limited number of murine cell models under *in vitro* culture conditions, which do not fully capture the metabolic heterogeneity of human HCC or the complexity of the tumor microenvironment. In addition, the metabolic signatures identified here have not yet been validated *in vivo*. Nevertheless, these limitations define important directions for future investigation. Extending this integrated endometabolomic and exometabolomic approach to additional murine and human HCC models, patient-derived systems, and *in vivo* settings will help determine the reproducibility and broader relevance of the metabolic signatures identified in this study. Namely, the validation of BCAA-derived ketoacids, pyroglutamate, and TCA-related metabolites across models of increasing aggressiveness could clarify whether these metabolic features are specific to Dt81Hepa1–6 cells or are reproducible across HCC models with different degrees of aggressiveness.

Finally, independent external validation would provide the strongest assessment of PLS-DA model generalizability. Given the available sample size, however, slitting the present dataset into separate training and validation sets would substantially reduce statistical power. The concordance between unsupervised PCA and PLS-DA, together with permutation testing, supports the robustness of the observed group discrimination, while validation in larger independent datasets remains warranted.

## Conclusions

This study demonstrates that integrated endometabolomic and exometabolomic ^1^H NMR profiling distinguishes primary hepatocytes from HCC cells and identifies metabolic features associated with the more aggressive phenotype of Dt81Hepa1–6 cells. In addition to established metabolic hallmarks of HCC, Dt81Hepa1–6 cells displayed a distinctive signature characterized by extracellular accumulation of BCAA-derived ketoacids, pyroglutamate, and TCA-related metabolites. These candidate metabolic features justify the need for further mechanistic investigation and validation in independent HCC models.

## Supporting information

S1 Table Experimental replication and sample numbers used for metabolomic analyses. The number of biological preparations, experimental replicates, and samples included in the endometabolomic and exometabolomic analyses is reported for each cell type. Exometabolome samples were obtained onward from the second primary hepatocyte preparation.
(TIFF)

S2 Table Univariate statistical analysis of the 15 intracellular metabolites with the highest PLS-DA VIP scores. Values are presented as mean ± SD for Dt81Hepa1–6 (DT), Hepa1–6 (H1-6), and primary hepatocytes (PH). Differences among cell types were assessed by one-way ANOVA followed by Fisher’s LSD post-hoc test. P-values were adjusted for multiple testing using the Benjamini-Hochberg false discovery rate (FDR) procedure. Effect sizes are reported as η^2^ with two-sided 95% confidence intervals. Significant Fisher’s LSD pairwise comparisons are also reported.
(TIFF)

S3 Table Two-way ANOVA of extracellular metabolites analyzed across cell types and sampling times. Effects of cell type (TYPE), sampling time (TIME), and their interaction (TYPE x TIME) are reported for the extracellular metabolites displayed in Fig 5B. P-values were adjusted for multiple testing using the Benjamini-Hochberg false discovery rate (FDR) procedure. Effect sizes are reported as ηp^2^ with two-sided 95% confidence intervals.
(TIFF)

S4 Table Univariate statistical analysis of the 15 extracellular metabolites with the highest PLS-DA VIP scores. Values are presented as mean ± SD for culture media from Dt81Hepa1–6 (DT), Hepa1–6 (H1-6), and primary hepatocytes (PH) cultures. Differences among groups were assessed by one-way ANOVA followed by Fisher’s LSD post-hoc test. P-values were adjusted for multiple testing using the Benjamini-Hochberg false discovery rate (FDR) procedure. Effect sizes are reported as η^2^ with two-sided 95% confidence intervals. Significant Fisher’s LSD pairwise comparisons are also reported.
(TIFF)

S1 Fig PCA biplots of endometabolic and exometabolic profiles. Biplots obtained from unsupervised Principal Component Analysis (PCA) of ^1^H NMR data from (A) polar cell extracts and (B) culture media. Samples correspond to primary hepatocytes (PH), Hepa1–6 (H1-6) and Dt81Hepa1–6 (DT). The metabolite loadings indicate the variables contributing to sample distribution in the PCA space.
(TIFF)

S2 Fig Heatmap and validation of the endometabolomic PLS-DA model. PLS-DA was performed on ^1^H NMR data from polar cell extracts of primary hepatocytes (PH), Hepa1–6 (H1-6) and Dt81Hepa1–6 (DT) cells collected at 24 h and 48h. (A) Heatmap showing relative metabolite abundance across samples, with samples represented in columns and metabolites in rows. (B) PLS-DA model performance parameters and permutation test based on 1000 permutations.
(TIFF)

S3 Fig PLS-DA score plot and heatmap of exometabolic profiles. PLS-DA was performed on ^1^H NMR data from culture media of primary hepatocytes (PH), Hepa1–6 (H1-6) and Dt81Hepa1–6 (DT) cultures collected at 24 h and 48h. (A) PLS-DA score plot showing the distribution of samples according to experimental group. (B) Heatmap showing relative metabolite abundance across samples, with samples represented in columns and metabolites in rows.
(TIFF)

S4 Fig Validation of the exometabolomic PLS-DA model. PLS-DA was performed on ^1^H NMR data from culture media of primary hepatocytes (PH), Hepa1–6 (H1-6) and Dt81Hepa1–6 (DT) cultures collected at 24 and 48h. (A) PLS-DA model performance parameters. (B) Permutation test based on 1000 permutations.
(TIFF)

S1 File Endometabolomic dataset. (TXT)

S2 File Exometabolomic dataset. (TXT)

## Abbreviations

ADP: Adenosine diphosphate
AMP: Adenosine monophosphate
ANOVA: Analysis of variance
ATP: Adenosine triphosphate
BCAA: Branched-Chain Amino Acid
DMEM: Dulbecco’s Modified Eagle Medium
DNA: Deoxyribonucleic Acid
DT: Dt81Hepa1–6
FID: Free Induction Decay
H1-6: Hepa1–6
HCC: Hepatocellular Carcinoma
LOOCV: Leave-One-Out Cross-Validation
MetOH: Methanol
NADH: reduced nicotinamide adenine dinucleotide
NMR: Nuclear Magnetic Resonance
PCA: Principal Component Analysis
PH: Primary Hepatocyte
PLS-DA: Partial Least Squares-Discriminant Analysis
TCA cycle: Tricarboxylic Acid cycle
TSP: sodium trimethylsilyl-(2,2,3,3-d_4_)-propionate
VIP: Variable Importance in Projection
